# Boosted Solar Light Absorbance in PdS_2_/PtS_2_ Vertical Heterostructures for Ultrathin Photovoltaic Devices

**DOI:** 10.1021/acsami.1c11245

**Published:** 2021-09-01

**Authors:** Lorenzo Bastonero, Giancarlo Cicero, Maurizia Palummo, Michele Re Fiorentin

**Affiliations:** † U Bremen Excellence Chair “Materials Design and Discovery” and Hybrid Materials Interfaces Group, Bremen Center for Computational Materials Science, University of Bremen, Am Fallturm 1, 28359 Bremen, Germany; ‡ Dipartimento di Scienza Applicata e Tecnologia, Politecnico di Torino, Corso Duca Degli Abruzzi 24, 10129 Torino, Italy; § Dipartimento di Fisica and INFN, Università di Roma “Tor Vergata”, Via Della Ricerca Scientifica 1, 00133 Roma, Italy; ∥ Center for Sustainable Future Technologies, Istituto Italiano di Tecnologia, Via Livorno 60, 10144 Torino, Italy; ⊥ Dipartimento di Fisica, Università Degli Studi Torino, Via Giuria 1, 10125 Torino, Italy

**Keywords:** photovoltaics, TMDs, van der Waals heterostructures, 2D materials, excitons

## Abstract

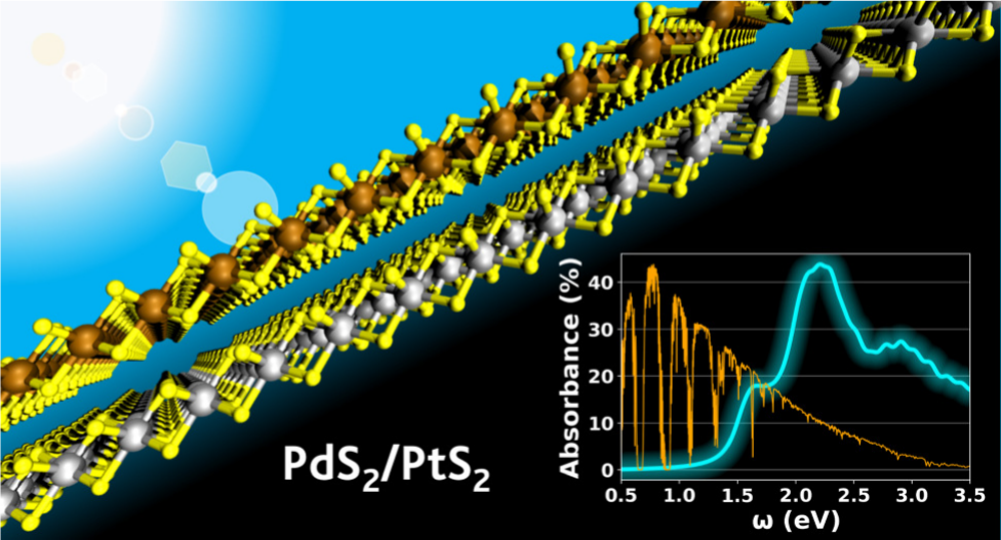

Transition-metal dichalcogenides (TMDs) represent a class of materials whose archetypes, such as MoS_2_ and WS_2_, possess exceptional electronic and optical properties and have been massively exploited in optoelectronic applications. The layered structure allows for their exfoliation to two-dimensional samples with atomic thickness (≲ 1 nm), promising for ultrathin, ultralight devices. In this work, by means of state-of-the-art *ab initio* many-body perturbation theory techniques, we focus on single-layer PdS_2_ and PtS_2_ and propose a novel van der Waals heterostructure with outstanding light absorbance, reaching up to 50% in the visible spectrum and yielding a maximum short-circuit current of 7.2 mA/cm^2^ under solar irradiation. The computed excitonic landscape predicts a partial charge separation between the two layers and the momentum-forbidden lowest-energy state increases the carrier diffusion length. Our results show that the employment of vertical heterostructures with less conventional TMDs, such as PdS_2_/PtS_2_, can greatly boost light absorbance and favor the development of more efficient, atomic-thin photovoltaic devices.

## 1 Introduction

Two-dimensional (2D) materials have elicited great attention over the last few decades thanks to their unique electronic and optical properties induced by their atomic-scale thickness.^1−3^ A plethora of materials are currently under investigation, and particular interest has been focused on transition-metal dichalcogenides (TMDs) in their monolayer form. TMDs can be easily exfoliated to single layers and allow for largely tunable optoelectronic properties,^4,5^ thanks to the plentiful possible combinations of transition metal and chalcogen species. For instance, band gaps of monolayer TMDs range from 0.5 to 7.0 eV^6^ and can be both direct and indirect. This wide range of electronic properties can be further enriched by devising vertical van der Waals heterostructures (vdWHs)^7,8^ between a monolayer TMD and another 2D material, such as graphene,^9,10^ black or blue phosphorene,^11,12^ hexagonal boron nitride,^13,14^ or a different TMD monolayer.^15,16^ vdWHs make it possible to realize devices that allow for the separation of the optically excited electrons and holes thanks to the valence and conduction band discontinuities of the two composing layers. Together with nanometer thickness and suitable band gaps, this feature makes TMD vdWHs appealing for the design of ultrathin, ultralight photovoltaic devices.^17^ In this respect, it is therefore necessary that the composing layers show significant light absorbance within the solar spectrum. For photon energies varying between 1.5 and 3 eV, widely studied TMDs show absorbance values that roughly range from 5 to 25% for MoS_2_^18^ to 20% for MoSe_2_ or 17% for WSe_2_ and WS_2_.^19^ Their vdWHs exhibit similar behaviors, for instance, reaching peak values of 10% in MoS_2_/WS_2_^20^ and in MoSe_2_/WSe_2_ or 15% in MoS_2_/WSe_2_.^21^ In combination with the incident AM1.5G solar flux Φ_s_(ω),^22^ the absorbance Abs(ω) determines the maximum short-circuit current that can be extracted from the solar cell as
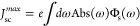
1assuming that all photogenerated charge carriers are collected. Common amorphous Si–H and GaAs solar cells in a single-pass configuration show *J*_sc_^max^ values which lie around 25 and 35 mA/cm^2^, for sample thicknesses above 1 μm, and quickly drop much below 1 mA/cm^2^ when their thickness is decreased to around 1 nm.^23^ In contrast, it has been predicted that a 1 nm-thick MoS_2_/WS_2_ vdWH displays a *J*_sc_^max^ ≃ 3.5 mA/cm^2^,^20^ the same value obtained in Si–H or GaAs cells about 1 order of magnitude thicker. The nanoscale thickness of the heterostructure allows for the design of ultralight devices with high power densities,^24^ even though the *J*_sc_^max^ remains low on an absolute scale. Higher short-circuit currents can be obtained by suitable arrangements of multiple heterostructures, albeit at the expense of the overall device thinness. Increasing the absorbance of the heterostructure, while keeping its thickness within ∼1 nm, is then of key importance for the development of new nanoscale photovoltaic devices that can introduce a paradigm shift in solar energy harvesting.

**Figure 1:**
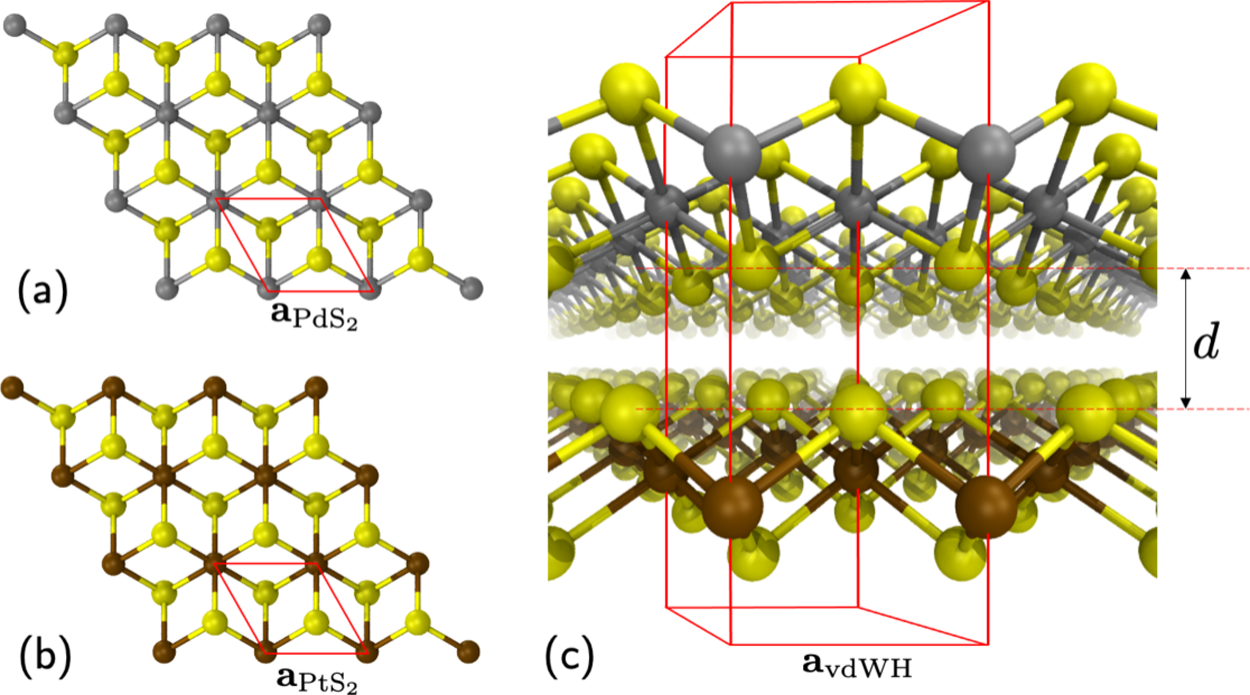
Monolayer PdS_2_ (a) and PtS_2_ (b) in the 1T phase. (c) Perspective view of the PdS_2_/PtS_2_ heterostructure with marked unit cell and lattice parameter *a*_vdW_ and layer separation *d*.

In this work, we focus on two less-studied single-layer TMDs which hold great potential, both as distinct monolayers and as part of a vdWH: PdS_2_ and PtS_2_. Single-layer PtS_2_ has been experimentally obtained from bulk PtS_2_^25,26^*via* exfoliation^27,28^ and reported to be in the 1T phase, Figure 1b. Monolayer PdS_2_ has been investigated in its 1T phase,^29^Figure 1a, and its exfoliation process from bulk crystals has been suggested.^30^ The lattice parameters and electronic properties of 1T-PtS_2_ and 1T-PdS_2_, as reported in the literature,^6^ suggest the possibility of realizing a low-strain vdWH with type-II band alignment. We therefore propose and investigate a novel 1T-PdS_2_/1T-PtS_2_ vdWH by employing state-of-the-art *ab initio* simulations, based on density functional theory (DFT) for geometry optimization and the GW-Bethe Salpeter^31,32^ approach for the study of optoelectronic properties.

## 2 Methods

### 2.1 DFT Calculations

All the DFT calculations reported in this work were performed using the Quantum ESPRESSO suite.^33−35^ For all systems, we employed the Perdew–Burke–Ernzerhof functional^36^ and fully relativistic, norm-conserving pseudopotentials^37^ on a uniform 4 × 4 × 1 Monkhorst–Pack *k*-point mesh.^38^ A kinetic energy cutoff of 70 Ry was adopted. We introduced a 20 Å vacuum region along *z*, the direction perpendicular to the layer planes, to ensure the decoupling of the periodic replicas. In the studied systems, spin–orbit coupling effects can be sizeable and were accounted for in all calculations by means of a fully spinorial treatment.^39,40^ Dispersion forces between the layers of the heterostructure were reproduced within the DFT-D3 semiempirical model.^41^ Structure relaxation was assumed at convergence when the maximum component of the residual forces on the ions was smaller than 10^–4^ Ry/Bohr.

### 2.2 Many-Body Perturbation Theory Calculations

The optimized structures were studied by means of many-body perturbation theory methods as implemented in the YAMBO code.^42,43^ The quasiparticle (QP) electronic structure was obtained within the non-self-consistent *G*_0_*W*_0_ approximation. A box cutoff along *z* was applied on the bare Coulomb potential. The inverse dielectric matrix, ε_GG′_^–1^, was obtained within the Godby-Needs plasmon-pole approximation model.^44,45^ As a convergence parameter, we adopted the QP band gap value at the Γ point and deemed satisfactory a convergence of this value within 50 meV. Convergence of the *G*_0_*W*_0_ calculations with respect to the simulation parameters is shown in Figure S1 in Supporting Information. Following the chosen convergence criterion, we chose a 20 × 20 × 1 *k*-point mesh for all structures.

For both PdS_2_ and PtS_2_, 2000 bands were included in the calculation of the dielectric function ε_GG′_^–1^ and a 20 Ry cutoff was set on the *G* vectors in ε^–1^.

Following ref^6^, we obtain the correction to the band gap due to the number of bands included in the correlation self-energy Σ_c_ by extrapolation to the limit of infinite bands, cf. Supporting Information, Figure S1c. For PdS_2_ and PtS_2_ monolayers, the calculations can then be performed with 1000 bands explicitly included in the sum and then corrected by the application of a rigid scissor operator of −0.12 eV. For the vdWH, we employ 4000 bands and 20 Ry in the calculation of the dielectric matrix and 1000 bands in Σ_c_, thanks to the adoption of Bruneval–Gonze terminators.^46^

Convergence of the BSE calculations with respect to the simulation parameters is shown in Figure S2 in Supporting Information. For PdS_2_ and PtS_2_, convergence is reached on a 30 × 30 × 1 grid, with 200 bands and a 2 Ry cutoff on the static screening and 6 valence +6 conduction bands in the BSE kernel. In the case of the vdWH, the number of included bands is doubled.

## 3 Results and Discussion

**Figure 2:**
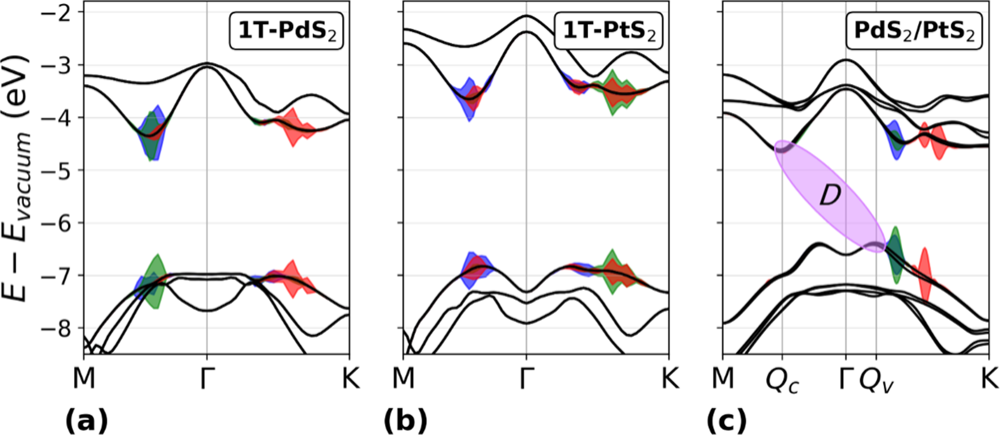
*G*_0_*W*_0_ band structures of (a) 1T-PdS_2_, (b) 1T-PtS_2_, and (c) PdS_2_/PtS_2_ vdWH aligned to the vacuum level. The shaded areas in red, green, and blue mark the excitonic weights of the R, G, and B states, respectively (see Figure 3). The violet ellipse in (c) indicates the indirect exciton *D* with finite momentum **q**_D_.

**Figure 3:**
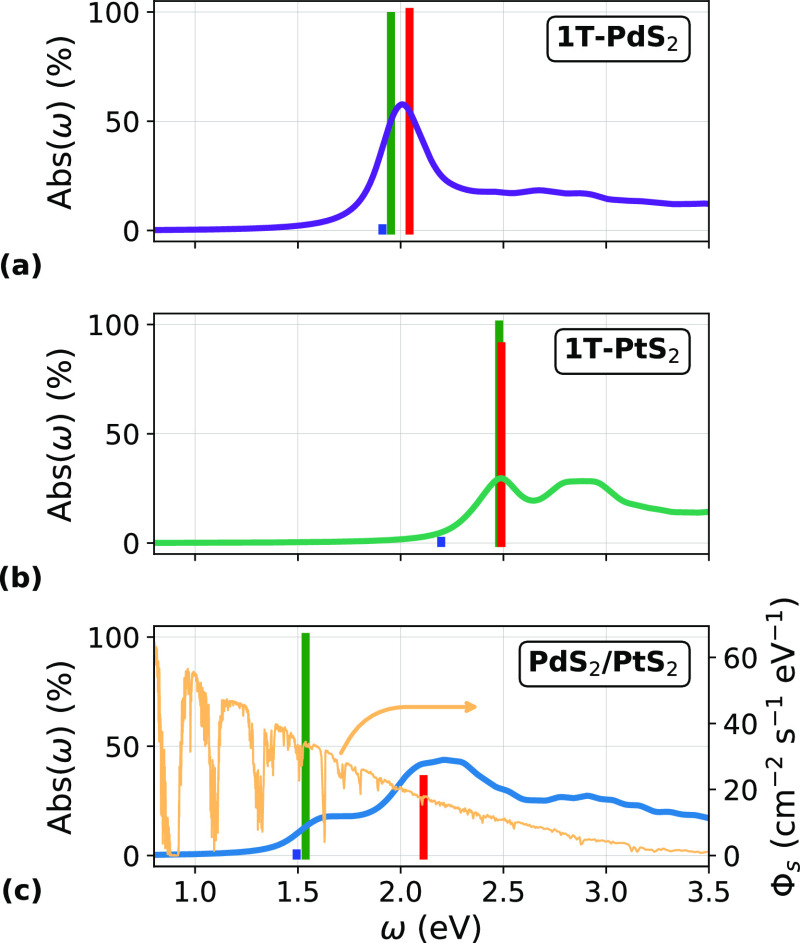
Absorbance Abs(ω) of (a) 1T-PdS_2_, (b) 1T-PtS_2_, and (c) PdS_2_/PtS_2_ vdWH. The vertical bars mark the position and relative intensities of the R, G, and B excitons, respectively. In (c), the AM1.5G solar spectrum Φ_s_(ω) is reported in light yellow.

From our DFT calculations, we compute the equilibrium lattice parameters of the isolated monolayers to be a_PdS2_ = 3.53 Å for 1T-PdS_2_ and a_PtS2_ = 3.56 Å for 1T-PtS_2_, in excellent agreement with the literature.^47^ By vertically stacking the two monolayers in the AA configuration,^48^ we obtain the vdWH geometry represented in Figure 1c, whose equilibrium lattice parameter results in *a*_vdWH_ = 3.57 Å and interlayer distance in *d* = 2.4 Å. The increase in the lattice parameter in the heterostructure can be traced back to the interaction between the layers and the reduced screening, as happens for multilayers of both PdS_2_ and PtS_2_.^49,50^ The resulting strain can be estimated as ε_i_ = (*a*_vdWH_ – *a*_i_)/*a*_i_ with i = PtS_2_ and PdS_2_. The highest value of ε is obtained for the PdS_2_ layer, which is subject to about 1.1% tensile strain with respect to its equilibrium geometry. Hence, the monolayers undergo a very mild strain, which does not significantly affect the electronic properties of the system (c.f. Figure S3a in Supporting Information). The equilibrium geometries of the isolated monolayers and the vdWH are used to compute the electronic band structure at the *G*_0_*W*_0_ level, as reported in Figure 2. On top of the *G*_0_*W*_0_ quasiparticle energies, we solve the Bethe–Salpeter equation to obtain the light absorption spectrum and excitonic properties, Figure 3. 1T-PdS_2_, Figure 2a, presents a top valence band (tVB) with a flattened parabolic structure around the Γ point and a conduction band minimum (CBm) at a *k*-point intermediate between *M* and Γ, resulting in an indirect band gap  and a direct gap  in correspondence with the CBm. The optical absorbance Abs(ω) is shown in Figure 3a, together with the lowest-energy exciton (blue) and the two brightest states (red and green) contributing to the absorption peak. Their energies and binding energies are reported in Table 1. The shaded areas in Figure 2a mark the excitonic weights of excitons B (blue), G (green), and R (red), projected on the QP band structure. From the shaded regions in Figure 2a, it is possible to notice that the strongest transitions contributing to the excitons, rather than occurring at high symmetry points, take place at *k*-points at which the valence and conduction bands present a similar slope along the *k*-path, giving rise to the band-nesting phenomenon.^51^ This feature is responsible for large divergencies in the joint density of states (jDOS) which, in turn, influences the material dielectric function, enhancing light absorption. We notice that the absorption peak of monolayer 1T-PdS_2_ falls around 2 eV and, remarkably, its absorbance reaches up to 55% in that range.

**Table 1:** Transition Energies *E*_λ_ [eV] and Exciton Binding Energies *E*_*b*_ [eV] of the Analyzed Excitons (B = Blue; R = Red; and G = Green)

	1T-PdS2			1T-PtS2			1T-PdS2/1T-PtS2		
	B	G	R	B	G	R	B	G	R
*E*_λ_	1.91	1.95	2.04	2.20	2.48	2.49	1.49	1.53	2.11
*E*_b_	0.8	0.8	0.7	0.9	0.6	0.6	0.7	0.6	0.1

1T-PtS_2_, Figure 2b, shows a peculiar Mexican-hat-shaped tVB, with a valence band maximum (VBM) and CBm midway between *M* and Γ. Although VBM and CBm are closer in *k*-space, the resulting band gap is nonetheless indirect, , while the direct gap is . The absorbance spectrum, Figure 3b, is blue-shifted with respect to 1T-PdS_2_: the absorption peak is due to two excitons (green and red), at around 2.5 eV, and a dark exciton (blue) is the lowest-energy state, about 30 meV below. The peak in absorbance of PtS_2_ is lower than the one of PdS_2_ and reaches about 30%. Such a difference in absorbance can be traced back to the different orbital character of the electronic states in the tVB and bCB of the two monolayers. In PdS_2_, we observe a sizeable participation of sulfur p orbitals in the tVB states, in contrast to PtS_2_, where S p and Pt d orbitals are almost equally contributing to the topmost valence band (c.f. Supporting Information Figures S4 and S5). The difference in the orbital character results in a stronger band-nesting for PdS_2_ which, consequently, increases the jDOS, as shown in Figure S6a,b. Moreover, we determined that the independent-particle electric dipole matrix elements *d*_cv_**k** of the transitions *v → c* at **k**, contributing to the absorption peak, are larger in PdS_2_ than in PtS_2_, c.f. Figure S6c. The larger jDOS and the stronger dipole elements thus cooperate to enhance the optical absorption of PdS_2_ with respect to PtS_2_.

In Figure 3b, at around 2.8 eV, it is also possible to notice a broader peak in the absorbance spectrum, originating from states, close in energy, whose main dipole transitions involve the bCB and tVB-1.

**Figure 4:**
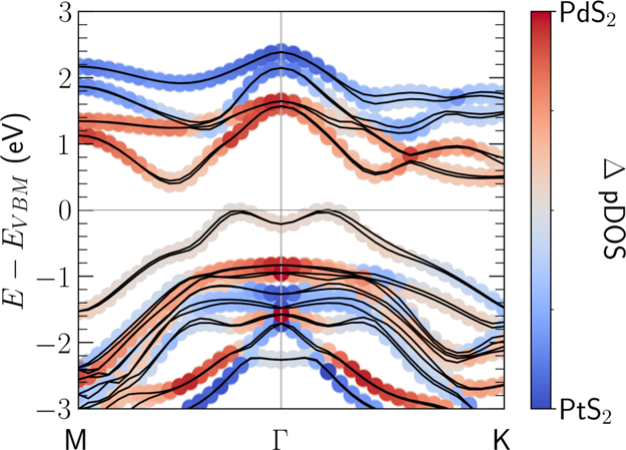
1T-PdS_2_/1T-PtS_2_ vdWH *k*-resolved projected density of states superimposed to the DFT band structure. Each state is colored according to the size of the contributions from PdS_2_ (red) and PtS_2_ (blue) orbitals.

Although less pronounced than in PdS_2_, the contribution from the p-orbitals of the outer S layers to the tVB of PtS_2_ is non-negligible, Figure S4. Therefore, in a vdWH, the tVB of each monolayer is significantly affected by the presence of the facing one, resulting in a vdWH tVB which shows sizeable contributions from both PdS_2_ and PtS_2_ valence band states. In Figure 4, we show the *k*-resolved density of states (DOS) of the 1T-PdS_2_/1T-PtS_2_ heterostructure, projected on the orbitals of the atoms composing the two layers. The bands along the *k*-path are colored according to the difference between the DOS contributions from the two layers, allowing us to identify on which layer the electronic states at various *k*-points are spatially localized. As mentioned, the vdWH tVB shows some degree of delocalization on both layers, while the bCB, being mostly composed of inner metal d-orbitals, is less affected by the interlayer interactions and clearly localized on 1T-PdS_2_. The G_0_W_0_-corrected band structure, aligned to the vacuum level, is reported in Figure 2c. In contrast to the isolated monolayers, in the vdWH case, the inversion symmetry is broken; hence, its band structure shows spin–orbit splitting. The band gap is indirect, *E*_vdWH_^*g*^ = 1.72 eV, between *k*-points **Q**_c_ and **Q**_v_, and notably smaller than each monolayer’s. The direct band gap is found to be *E*_vdWH_^*g*,dir^ = 2.17 eV in correspondence with **Q**_v_. The electronic band gap reduction is an expected feature linked to the type-II alignment of the single TMD band structures, cf. Figure 2a,b, and the interaction between the two layers, which determines an upshift of the top valence band, and the increased electronic screening. This similarly entails a reduction in the optical band gap, as shown in Figure 3c. The absorption onset red-shifts to about 1.5 eV and stems from a bright exciton (labeled “green”, G) at 1.53 eV, c.f. Table 1. This exciton is almost degenerate in energy with the lowest-energy, dark state (blue, B), whose constituting transitions take place at the same *k*-points as the green exciton but connect states with the opposite spin.

By means of eq 1 and the absorbance spectra in Figure 3, we can compute the maximum short-circuit currents that can be extracted from the monolayers and from the vdWH, in the idealized situation of total carrier collection at the electrodes. 1T-PtS_2_ has a higher-energy absorption edge and a moderate absorption, resulting in *J*_sc_^max^ = 1.9 mA/cm^2^, while 1T-PdS_2_ shows a lower absorption onset and outstanding absorbance values so that its maximum short-circuit current reaches 5.4 mA/cm^2^. The vdWH shows less pronounced absorbance peaks than 1T-PdS_2_; however, the optical gap falls at lower energy, optimizing sunlight absorption in the visible range. Hence, the short-circuit current of the heterostructure reaches 7.2 mA/cm^2^, slightly lower than the sum of the *J*_sc_^max^ of the isolated monolayers. The predicted value of maximum short-circuit current is exceptionally high and can largely boost the efficiency of a device made with such a vdWH. As a comparison, the obtained maximum short-circuit current is twice the predicted value in MoS_2_/WS_2_ vdWH^20^ and can be obtained with amorphous Si–H samples about 2 orders of magnitude thicker.

**Figure 5:**
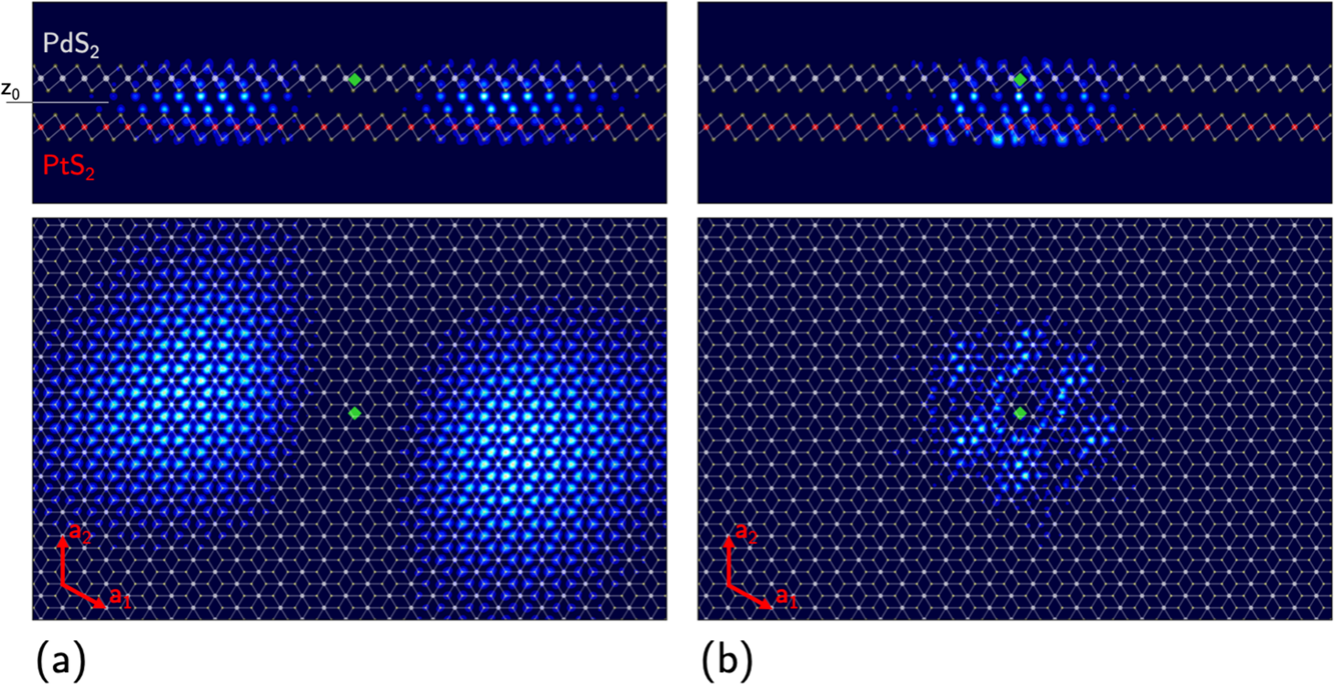
Side (upper panels) and top (lower panels) views of the square modulus excitonic wave function |Ψ_λ_(***r***_h_|**r**_e_ = ***r*^′^**)|^2^ for states λ = *D* (a) and λ = *G* (b). In both (a,b), the electron is marked by the green diamond and kept fixed on PdS_2_.

The brightest excitons, red and green, which induce the peaks in the absorption spectrum of the vdWH, vertically excite electrons and holes at *k*-points away from the CBm and VBM, respectively. In mono- and few-layer TMDs, intraband relaxation is very fast (≲500 fs);^52,53^ therefore, we can expect a rapid relaxation of the optically active excitons to the dark, indirect exciton *D*, involving states at the CBm and VBM, violet ellipse in Figure 2c. The relaxation to this exciton, with finite momentum ***q***_D_ = **Q**_c_ – **Q**_v_, is energetically favorable, since *E*_D_ = 1.15 eV, smaller than the dark direct exciton *B*. Being momentum-forbidden, the radiative recombination of state *D* is suppressed and, consequently, its lifetime is largely enhanced with respect to bright excitons.^54^ Electron–hole recombination is therefore slowed down, and the carrier diffusion length increased. Hence, due to the indirect gap in the 1T-PdS_2_/1T-PdS_2_ heterostructure, we expect carrier diffusion lengths longer than 20 μm, as recently measured in the direct-gap WS_2_/WSe_2_ vdWH.^55^ The expected sizeable diffusion length will then contribute to enhance the actual short-circuit current of the real device, by increasing the collection probability, thus positively impacting on the efficiency of the cell. These efficiency boosting factors, that is, very large absorbance and increased diffusion length, can then counterbalance the limited charge separation due to the mixed PdS_2_–PtS_2_ states in the tVB, c.f. Figure 4, that could also act as recombination centers. Indeed, the optically excited electrons will populate states which are well spatially localized on the PdS_2_ layer, while the holes will still present a sizeable delocalization on both monolayers. In Figure 5a, we show the square modulus of the *D* exciton wave function as a function of the hole position |Ψ_D_(**r**_h_|**r**_e_ = ***r***^′^)|^2^, when the electron is fixed at ***r*′** on the PdS_2_ layer, in a region with maximum probability density. The electron is marked by the green diamond on the PdS_2_ layer. The electron and hole show a peculiar in-plane spatial separation, due to the electronic orbitals involved in *D*. As a comparison, in Figure 5b, we report the square modulus of the bright, *G* exciton wave function, with fixed electron in the same position. It is evident that the *G* and *D* excitons are not spatially indirect between the two layers, and their wave functions involve both PdS_2_ and PtS_2_. From Figure 5a, it is possible to notice that the hole is fairly delocalized on both layers and it is possible to obtain the probability of finding the hole on the PtS_2_ layer as ∫_z_h_<*z*_0__d*r*_h_|Ψ_D_(*r*_h_|*r*_e_ = *r*^′^)|^2^ ≃ 50%, where *z*_0_ defines the intermediate plane between the two monolayers. This provides us with an estimate of the charge separation probability, given that the hole distribution varies little when the electron position is moved in the region of sizeable probability density, concentrated on PdS_2_. The estimated charge separation is not complete, however, its magnitude, combined with the encouraging properties previously highlighted, can be nonetheless adequate for viable applications of the proposed vdWH. Moreover, it has been proven that spatially indirect excitons inducing interlayer charge separation can be enhanced by applying strain^56,57^ to the heterostructure or controlled through electric fields,^58^ thus conceivably leaving room for further efficiency improvements.

## 4 Conclusions

The field of nanoscale photovoltaic devices is still in open development and appears alluring for its potential to compete with conventional systems. The intrinsic advantages of ultrathin, ultralight devices urge the research activities toward improved efficiency and design. The employment of monolayer TMDs in heterostructures has been proposed and investigated, with most attention paid to group-VI TMDs, such as MoS_2_ and WS_2_, which have provided encouraging results. A key factor for choosing TMDs for this type of application resides in their large light-electron coupling, which results in sizeable light absorbance. Our work unveiled the remarkable properties of two, less common, TMDs, PdS_2_ and PtS_2_, which can be positively employed in the domain of photovoltaics. In particular, the uncommon absorbance of 1T-PdS_2_ is inherited by the low-strain vdWH with PtS_2_, which also shows a very favorable optical gap at around 1.5 eV. This translates into exceptionally high maximum short-circuit currents, *J*_sc_^max^ = 7.2 mA/cm^2^, about twice the values previously reported for MoS_2_/WS_2_ vdWH. Although the predicted charge separation is partial, the high absorbance, together with the extended carrier diffusion length, due to the indirect electronic band gap, can turn out very advantageous for improving the efficiency of nanometer-thick photovoltaic devices.

## Supporting Information Available

*G*_0_*W*_0_ and BSE convergence tests, details on PdS_2_ and PtS_2_ monolayer optical absorbance, and selected wave functions (PDF)

## References

[ref1] Bhimanapati G. R.; Lin Z.; Meunier V.; Jung Y.; Cha J.; Das S.; Xiao D.; Son Y.; Strano M. S.; Cooper V. R.; Liang L.; Louie S. G.; Ringe E.; Zhou W.; Kim S. S.; Naik R. R.; Sumpter B. G.; Terrones H.; Xia F.; Wang Y.; Zhu J.; Akinwande D.; Alem N.; Schuller J. A.; Schaak R. E.; Terrones M.; Robinson J. A. Recent Advances in Two-Dimensional Materials Beyond Graphene. ACS Nano 2015, 9, 11509–11539. 10.1021/acsnano.5b05556. 26544756

[ref2] Tan T.; Jiang X.; Wang C.; Yao B.; Zhang H. 2D Material Optoelectronics for Information Functional Device Applications: Status and Challenges. Adv. Sci. 2020, 7, 2000058 10.1002/advs.202000058. PMC7284198 32537415

[ref3] Kang S.; Lee D.; Kim J.; Capasso A.; Kang H. S.; Park J.-W.; Lee C.-H.; Lee G.-H. 2D Semiconducting Materials for Electronic and Optoelectronic Applications: Potential and Challenge. 2D Materials 2020, 7, 022003 10.1088/2053-1583/ab6267.

[ref4] Huang H. H.; Fan X.; Singh D. J.; Zheng W. T. Recent Progress of TMD Nanomaterials: Phase Transitions and Applications. Nanoscale 2020, 12, 1247–1268. 10.1039/c9nr08313h. 31912836

[ref5] Manzeli S.; Ovchinnikov D.; Pasquier D.; Yazyev O. V.; Kis A. 2D Transition Metal Dichalcogenides. Nat. Rev. Mater. 2017, 2, 17033 10.1038/natrevmats.2017.33.

[ref6] Rasmussen F. A.; Thygesen K. S. Computational 2D Materials Database: Electronic Structure of Transition-Metal Dichalcogenides and Oxides. J. Phys. Chem. C 2015, 119, 13169–13183. 10.1021/acs.jpcc.5b02950.

[ref7] Liu Y.; Weiss N. O.; Duan X.; Cheng H.-C.; Huang Y.; Duan X. Van Der Waals Heterostructures and Devices. Nat. Rev. Mater. 2016, 1, 16042 10.1038/natrevmats.2016.42.

[ref8] Geim A. K.; Grigorieva I. V. Van Der Waals Heterostructures. Nature 2013, 499, 419–425. 10.1038/nature12385. 23887427

[ref9] Li C.; Cao Q.; Wang F.; Xiao Y.; Li Y.; Delaunay J.-J.; Zhu H. Engineering Graphene and TMDs Based Van Der Waals Heterostructures for Photovoltaic and Photoelectrochemical Solar Energy Conversion. Chem. Soc. Rev. 2018, 47, 4981–5037. 10.1039/c8cs00067k. 29736528

[ref10] Azadmanjiri J.; Srivastava V. K.; Kumar P.; Sofer Z.; Min J.; Gong J. Graphene-Supported 2D Transition Metal Dichalcogenide Van Der Waals Heterostructures. Appl. Mater. Today 2020, 19, 100600 10.1016/j.apmt.2020.100600.

[ref11] Peng Q.; Wang Z.; Sa B.; Wu B.; Sun Z. Electronic Structures and Enhanced Optical Properties of Blue Phosphorene/Transition Metal Dichalcogenides Van Der Waals Heterostructures. Sci. Rep. 2016, 6, 31994 10.1038/srep31994. 27553787 PMC4995501

[ref12] Maniyar A.; Choudhary S. Visible Region Absorption in TMDs/phosphorene Heterostructures for Use in Solar Energy Conversion Applications. RSC Adv. 2020, 10, 31730–31739. 10.1039/d0ra05810f. PMC9056560 35518129

[ref13] Latini S.; Winther K. T.; Olsen T.; Thygesen K. S. Interlayer Excitons and Band Alignment in MoS2/hBN/WSe2 Van Der Waals Heterostructures. Nano Lett. 2017, 17, 938–945. 10.1021/acs.nanolett.6b04275. 28026961

[ref14] Jadczak J.; Kutrowska-Girzycka J.; Bieniek M.; Kazimierczuk T.; Kossacki P.; Schindler J. J.; Debus J.; Watanabe K.; Taniguchi T.; Ho C. H.; Wójs A.; Hawrylak P.; Bryja L. Probing Negatively Charged and Neutral Excitons in MoS2/hBN and hBN/MoS2/hBN Van Der Waals Heterostructures. Nanotechnology 2021, 32, 145717 10.1088/1361-6528/abd507. 33463532

[ref15] Huo N.; Yang Y.; Li J. Optoelectronics Based on 2D TMDs and Heterostructures. J. Semiconduct. 2017, 38, 031002 10.1088/1674-4926/38/3/031002.

[ref16] Saha D.; Varghese A.; Lodha S. Atomistic Modeling of Van Der Waals Heterostructures With Group-6 and Group-7 Monolayer Transition Metal Dichalcogenides for Near Infrared/Short-Wave Infrared Photodetection. ACS Appl. Nano Mater. 2020, 3, 820–829. 10.1021/acsanm.9b02342.

[ref17] Furchi M. M.; Höller F.; Dobusch L.; Polyushkin D. K.; Schuler S.; Mueller T. Device Physics of Van Der Waals Heterojunction Solar Cells. npj 2D Mater. Appl. 2018, 2, 3 10.1038/s41699-018-0049-3.

[ref18] Li Y.; Chernikov A.; Zhang X.; Rigosi A.; Hill H. M.; van der Zande A. M.; Chenet D. A.; Shih E.-M.; Hone J.; Heinz T. F. Measurement of the optical dielectric function of monolayer transition-metal dichalcogenides:MoS2,MoSe2,WS2, andWSe2. Phys. Rev. B: Condens. Matter Mater. Phys. 2014, 90, 205422 10.1103/physrevb.90.205422.

[ref19] Wurstbauer U.; Miller B.; Parzinger E.; Holleitner A. W. Light-matter interaction in transition metal dichalcogenides and their heterostructures. J. Phys. D: Appl. Phys. 2017, 50, 173001 10.1088/1361-6463/aa5f81.

[ref20] Bernardi M.; Palummo M.; Grossman J. C. Extraordinary Sunlight Absorption and One Nanometer Thick Photovoltaics Using Two-Dimensional Monolayer Materials. Nano Lett. 2013, 13, 3664–3670. 10.1021/nl401544y. 23750910

[ref21] Flöry N.; Jain A.; Bharadwaj P.; Parzefall M.; Taniguchi T.; Watanabe K.; Novotny L. A WSe2/MoSe2 Heterostructure Photovoltaic Device. Appl. Phys. Lett. 2015, 107, 123106 10.1063/1.4931621.

[ref22] National Renewable Energy Laboratory. Solar Spectra. http://rredc.nrel.gov/solar/spectra/am1.5/ (accessed 30 March 2021).

[ref23] Andreani L. C.; Bozzola A.; Kowalczewski P.; Liscidini M.; Redorici L. Silicon Solar Cells: Toward the Efficiency Limits. Adv. Phys.: X 2019, 4, 1548305 10.1080/23746149.2018.1548305.

[ref24] Jariwala D.; Davoyan A. R.; Wong J.; Atwater H. A. Van Der Waals Materials for Atomically-Thin Photovoltaics: Promise and Outlook. ACS Photonics 2017, 4, 2962–2970. 10.1021/acsphotonics.7b01103.

[ref25] Furuseth S.; Selte K.; Kjekshus A.; Gronowitz S.; Hoffman R. A.; Westerdahl A. Redetermined Crystal Structures of NiTe2, PdTe2, PtS2, PtSe2, and PtTe2. Acta Chem. Scand. 1965, 19, 257–258. 10.3891/acta.chem.scand.19-0257.

[ref26] Hulliger F. Electrical Properties of Some Nickel-Group Chalcogenides. J. Phys. Chem. Solids 1965, 26, 639–645. 10.1016/0022-3697(65)90140-x.

[ref27] Zhao Y.; Qiao J.; Yu P.; Hu Z.; Lin Z.; Lau S. P.; Liu Z.; Ji W.; Chai Y. Extraordinarily Strong Interlayer Interaction in 2D Layered PtS2. Adv. Mater. 2016, 28, 2399–2407. 10.1002/adma.201504572. 26833689

[ref28] Tang C. Y.; Cheng P. K.; Wang X. Y.; Ma S.; Long H.; Tsang Y. H. Size-Dependent Nonlinear Optical Properties of Atomically Thin PtS2 Nanosheet. Opt. Mater. 2020, 101, 109694 10.1016/j.optmat.2020.109694.

[ref29] Miró P.; Ghorbani-Asl M.; Heine T. Two Dimensional Materials Beyond MoS2: Noble-Transition-Metal Dichalcogenides. Angew. Chem., Int. Ed. 2014, 53, 3015–3018. 10.1002/anie.201309280. 24554594

[ref30] Wang Y.; Li Y.; Chen Z. Not your familiar two dimensional transition metal disulfide: structural and electronic properties of the PdS2monolayer. J. Mater. Chem. C 2015, 3, 9603 10.1039/c5tc01345c.

[ref31] Onida G.; Reining L.; Rubio A. Electronic excitations: density-functional versus many-body Green’s-function approaches. Rev. Mod. Phys. 2002, 74, 601–659. 10.1103/revmodphys.74.601.

[ref32] Martin R.; Reining L.; Ceperley D. Interacting Electrons: Theory and Computational Approaches; Cambridge University Press, 2016.

[ref33] Giannozzi P.; Baroni S.; Bonini N.; Calandra M.; Car R.; Cavazzoni C.; Ceresoli D.; Chiarotti G. L.; Cococcioni M.; Dabo I.; Dal Corso A.; de Gironcoli S.; Fabris S.; Fratesi G.; Gebauer R.; Gerstmann U.; Gougoussis C.; Kokalj A.; Lazzeri M.; Martin-Samos L.; Marzari N.; Mauri F.; Mazzarello R.; Paolini S.; Pasquarello A.; Paulatto L.; Sbraccia C.; Scandolo S.; Sclauzero G.; Seitsonen A. P.; Smogunov A.; Umari P.; Wentzcovitch R. M. QUANTUM ESPRESSO: A Modular and Open-Source Software Project for Quantum Simulations of Materials. J. Phys.: Condens. Matter 2009, 21, 395502 10.1088/0953-8984/21/39/395502. 21832390

[ref34] Giannozzi P.; Andreussi O.; Brumme T.; Bunau O.; Buongiorno Nardelli M.; Calandra M.; Car R.; Cavazzoni C.; Ceresoli D.; Cococcioni M.; Colonna N.; Carnimeo I.; Dal Corso A.; de Gironcoli S.; Delugas P.; DiStasio R. A.; Ferretti A.; Floris A.; Fratesi G.; Fugallo G.; Gebauer R.; Gerstmann U.; Giustino F.; Gorni T.; Jia J.; Kawamura M.; Ko H.-Y.; Kokalj A.; Küçükbenli E.; Lazzeri M.; Marsili M.; Marzari N.; Mauri F.; Nguyen N. L.; Nguyen H.-V.; Otero-de-la-Roza A.; Paulatto L.; Poncé S.; Rocca D.; Sabatini R.; Santra B.; Schlipf M.; Seitsonen A. P.; Smogunov A.; Timrov I.; Thonhauser T.; Umari P.; Vast N.; Wu X.; Baroni S. Advanced Capabilities for Materials Modelling With Quantum ESPRESSO. J. Phys.: Condens. Matter 2017, 29, 465901 10.1088/1361-648x/aa8f79. 29064822

[ref35] Giannozzi P.; Baseggio O.; Bonfà P.; Brunato D.; Car R.; Carnimeo I.; Cavazzoni C.; de Gironcoli S.; Delugas P.; Ferrari Ruffino F.; Ferretti A.; Marzari N.; Timrov I.; Urru A.; Baroni S. QuantumESPRESSO toward the exascale. J. Chem. Phys. 2020, 152, 154105 10.1063/5.0005082. 32321275

[ref36] Perdew J. P.; Burke K.; Ernzerhof M. Generalized Gradient Approximation Made Simple. Phys. Rev. Lett. 1996, 77, 3865–3868. 10.1103/physrevlett.77.3865. 10062328

[ref37] Hamann D. Optimized Norm-Conserving Vanderbilt Pseudopotentials. Phys. Rev. B: Condens. Matter Mater. Phys. 2013, 88, 085117 10.1103/physrevb.88.085117.

[ref38] Monkhorst H. J.; Pack J. D. Special Points for Brillouin-Zone Integrations. Phys. Rev. B: Condens. Matter Mater. Phys. 1976, 13, 5188–5192. 10.1103/physrevb.13.5188.

[ref39] Corso A. D.; Conte A. M. Spin-Orbit Coupling With Ultrasoft Pseudopotentials: Application to Au and Pt. Phys. Rev. B: Condens. Matter Mater. Phys. 2005, 71, 115106 10.1103/physrevb.71.115106.

[ref40] Marsili M.; Molina-Sánchez A.; Palummo M.; Sangalli D.; Marini A. Spinorial formulation of the GW -BSE equations and spin properties of excitons in two-dimensional transition metal dichalcogenides. Phys. Rev. B 2021, 103, 155152 10.1103/physrevb.103.155152.

[ref41] Grimme S. Semiempirical GGA-type Density Functional Constructed With a Long-Range Dispersion Correction. J. Comput. Chem. 2006, 27, 1787–1799. 10.1002/jcc.20495. 16955487

[ref42] Marini A.; Hogan C.; Grüning M.; Varsano D. Yambo: An Ab Initio Tool for Excited State Calculations. Comput. Phys. Commun. 2009, 180, 1392 10.1016/j.cpc.2009.02.003.

[ref43] Sangalli D.; Ferretti A.; Miranda H.; Attaccalite C.; Marri I.; Cannuccia E.; Melo P.; Marsili M.; Paleari F.; Marrazzo A.; Prandini G.; Bonfà P.; Atambo M. O.; Affinito F.; Palummo M.; Molina-Sánchez A.; Hogan C.; Grüning M.; Varsano D.; Marini A. Many-Body Perturbation Theory Calculations Using the Yambo Code. J. Phys.: Condens. Matter 2019, 31, 325902 10.1088/1361-648x/ab15d0. 30943462

[ref44] Godby R. W.; Needs R. J. Metal-Insulator Transition in Kohn-Sham Theory and Quasiparticle Theory. Phys. Rev. Lett. 1989, 62, 1169–1172. 10.1103/physrevlett.62.1169. 10039594

[ref45] Rojas H. N.; Godby R. W.; Needs R. J. Space-Time Method forAb InitioCalculations of Self-Energies and Dielectric Response Functions of Solids. Phys. Rev. Lett. 1995, 74, 1827–1830. 10.1103/physrevlett.74.1827. 10057767

[ref46] Bruneval F.; Gonze X. Accurate GW Self-Energies in a Plane-Wave Basis Using Only a Few Empty States: Towards Large Systems. Phys. Rev. B: Condens. Matter Mater. Phys. 2008, 78, 085125 10.1103/physrevb.78.085125.

[ref47] Sajjad M.; Singh N.; Schwingenschlögl U. Strongly Bound Excitons in Monolayer PtS2 and PtSe2. Appl. Phys. Lett. 2018, 112, 043101 10.1063/1.5010881.

[ref48] By directly checking the stability of different stacking patterns, we identified the AA structure to be the most stable.

[ref49] Villaos R. A. B.; Crisostomo C. P.; Huang Z.-Q.; Huang S.-M.; Padama A. A. B.; Albao M. A.; Lin H.; Chuang F.-C. Thickness Dependent Electronic Properties of Pt Dichalcogenides. npj 2D Mater. Appl. 2019, 3, 2 10.1038/s41699-018-0085-z.

[ref50] Ahmad S. Strain and Electric Field Dependent Variation in Electronic and Thermoelectric Properties of PtS2. Results Phys. 2020, 17, 103088 10.1016/j.rinp.2020.103088.

[ref51] Carvalho A.; Ribeiro R. M.; Castro Neto A. H. Band Nesting and the Optical Response of Two-Dimensional Semiconducting Transition Metal Dichalcogenides. Phys. Rev. B: Condens. Matter Mater. Phys. 2013, 88, 115205 10.1103/physrevb.88.115205.

[ref52] Shi H.; Yan R.; Bertolazzi S.; Brivio J.; Gao B.; Kis A.; Jena D.; Xing H. G.; Huang L. Exciton Dynamics in Suspended Monolayer and Few-Layer MoS22D Crystals. ACS Nano 2013, 7, 1072–1080. 10.1021/nn303973r. 23273148

[ref53] Sohier T.; Campi D.; Marzari N.; Gibertini M. Mobility of Two-Dimensional Materials From First Principles in an Accurate and Automated Framework. Phys. Rev. Mater. 2018, 2, 114010 10.1103/physrevmaterials.2.114010.

[ref54] Kuroda T.; Hoshi Y.; Masubuchi S.; Okada M.; Kitaura R.; Watanabe K.; Taniguchi T.; Machida T. Dark-state impact on the exciton recombination of WS2 monolayers as revealed by multi-timescale pump-probe spectroscopy. Phys. Rev. B 2020, 102, 195407 10.1103/physrevb.102.195407.

[ref55] Jin C.; Kim J.; Utama M. I. B.; Regan E. C.; Kleemann H.; Cai H.; Shen Y.; Shinner M. J.; Sengupta A.; Watanabe K.; Taniguchi T.; Tongay S.; Zettl A.; Wang F. Imaging of pure spin-valley diffusion current in WS2-WSe2heterostructures. Science 2018, 360, 893–896. 10.1126/science.aao3503. 29798880

[ref56] Mueller T.; Malic E. Exciton Physics and Device Application of Two-Dimensional Transition Metal Dichalcogenide Semiconductors. npj 2D Mater. Appl. 2018, 2, 29 10.1038/s41699-018-0074-2.

[ref57] Re Fiorentin M.; Cicero G.; Palummo M. Spatially Indirect Excitons in Black and Blue Phosphorene Double Layers. Phys. Rev. Mater. 2020, 4, 074009 10.1103/physrevmaterials.4.074009.

[ref58] Jauregui L. A.; Joe A. Y.; Pistunova K.; Wild D. S.; High A. A.; Zhou Y.; Scuri G.; De Greve K.; Sushko A.; Yu C.-H.; Taniguchi T.; Watanabe K.; Needleman D. J.; Lukin M. D.; Park H.; Kim P. Electrical Control of Interlayer Exciton Dynamics in Atomically Thin Heterostructures. Science 2019, 366, 870–875. 10.1126/science.aaw4194. 31727834

