# Supplementary material for: Boosted Solar Light Absorbance in PdS2/PtS2 Vertical Heterostructures for Ultrathin Photovoltaic Devices
Source: ACS Appl Mater Interfaces. 2021 Sep 1;13(36):43615–21. doi: 10.1021/acsami.1c11245 (PMC8447185; doi:10.1021/acsami.1c11245)
Supplement: Supplementary file 1 — am1c11245_si_001.pdf [file am1c11245_si_001.pdf]

Supporting Information for

Boosted solar light absorbance in PdS_2/PtS_2 vertical heterostructures for ultrathin photovoltaic devices

Lorenzo Bastonero[a,b], Giancarlo Cicero[c], Maurizia Palummo[d] and Michele Re Fiorentin[e,*]

[a] *Dipartimento di Fisica, Università degli studi Torino, via Giuria 1, 10125, Torino, Italy*
[b] *U Bremen Excellence Chair "Materials design and discovery" and Hybrid Materials Interfaces Group, Bremen Center for Computational Materials Science, University of Bremen, Am Fallturm 1, 28359 Bremen, Germany*
[c] *Dipartimento di Scienza Applicata e Tecnologia, Politecnico di Torino, corso Duca degli Abruzzi 24, 10129 Torino, Italy*
[d] *Dipartimento di Fisica and INFN, Università di Roma "Tor Vergata", via della Ricerca Scientifica 1, 00133, Roma, Italy*
[e] *Center for Sustainable Future Technologies, Istituto Italiano di Tecnologia, via Livorno 60, 10144 Torino, Italy*

[*] michele.refiorentin@iit.it

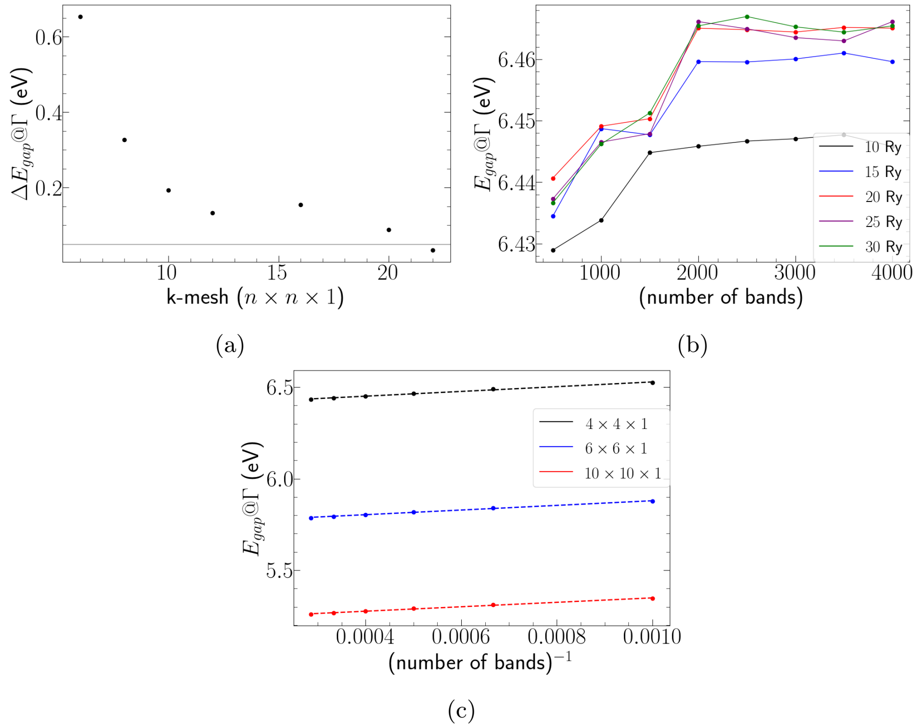

Figure S1: Convergence of GW calculations with respect to the k-point grid (a) and the dielectric screening (b). In (c) we show the extrapolation procedure to obtain the correction due to the number of included bands in Σ_c. Reported values are for PtS_2.

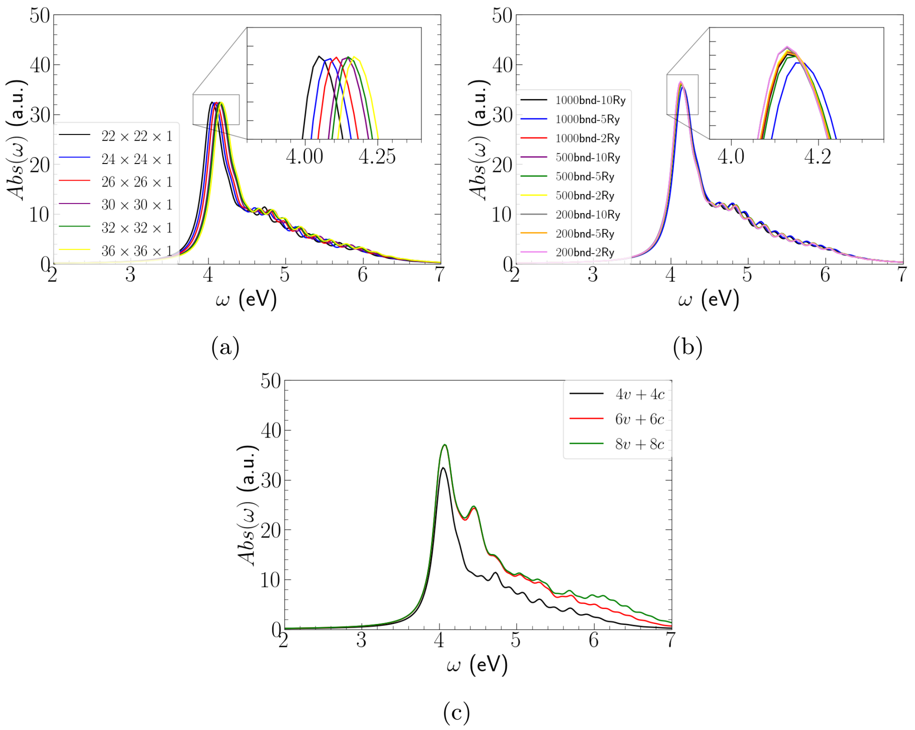

Figure S2: Convergence of BSE calculations with respect to the k-point grid (a), the static screening parameters (b) and the number of bands included in the kernel (c). Reported values are for PtS_2.

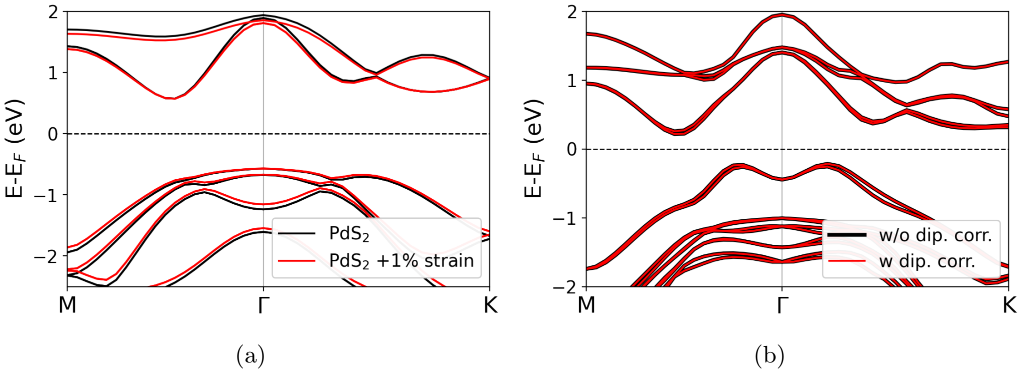

Figure S3: (a) DFT electronic bandstructure of relaxed monolayer Pd_2 (black lines) and monolayer Pd_2 under 1% biaxial strain (red lines). (b) DFT electronic bandstructure of the PdS_2/PtS_2 vdWH computed with (red lines) and without (black lines) dipole correction along the out-of-plane direction, as implemented in [1].

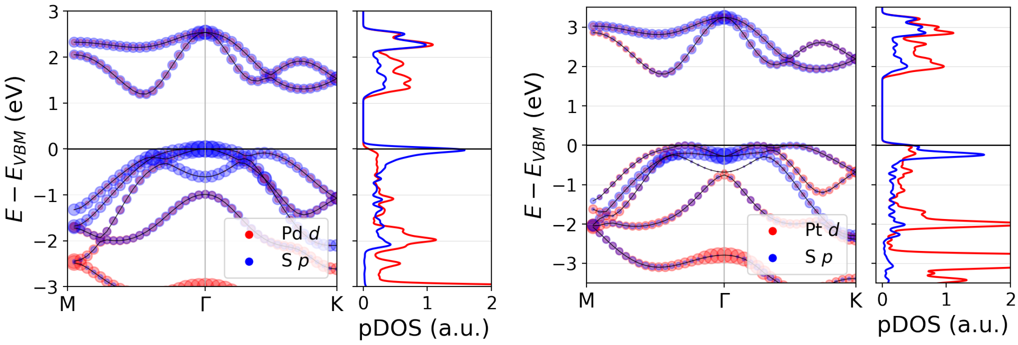

(a) (b)

Figure S4: pDOS and kpDOS of PdS_2 (a) and PtS_2 (b) as obtained from DFT calculations. Contributions from metal d-orbitals and sulfur p-orbitals are reported in red and blue, respectively.

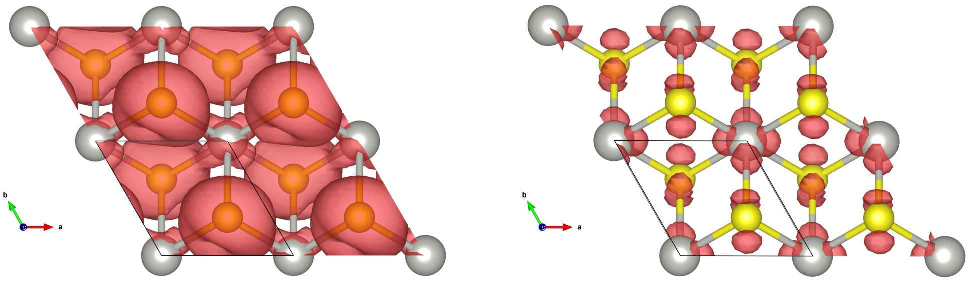

(a) (b)

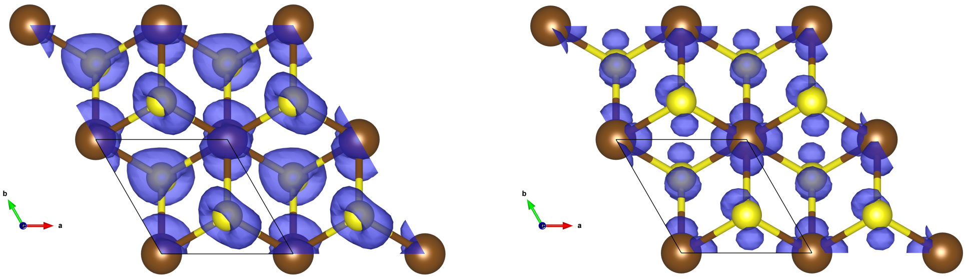

(c) (d)

Figure S5: (a), (b) Square moduli of the wavefunctions of the tVB and bCB states, respectively, involved in the G exciton for PdS_2. (c), (d) Square moduli of the wavefunctions of the tVB and bCB states, respectively, involved in the G exciton for PtS_2.

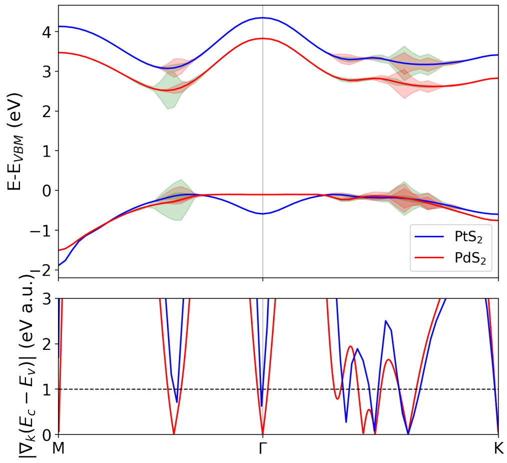

(a)

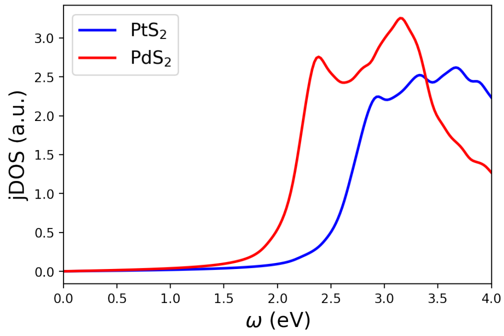

(b)

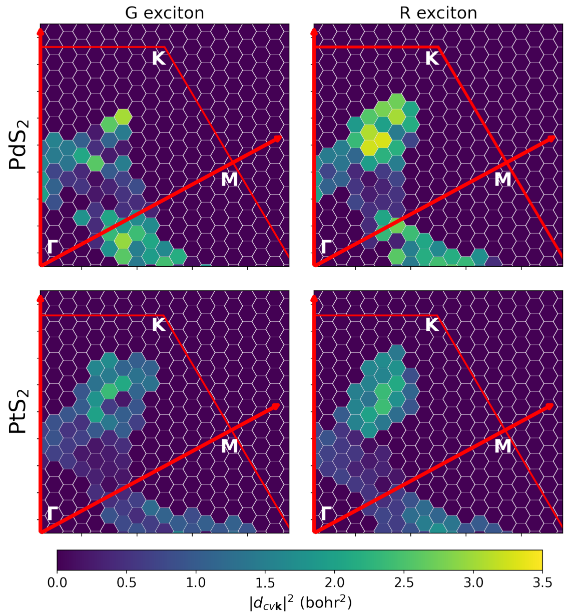

(c)

Figure S6: (a) Top panel: QP bandstructures of PdS_2 (red lines) and PtS_2 (blue lines). Lower panel: gradient along the Brillouin zone path of the difference between the bCB and tVB energies. In correspondence with small values (<1) the band-nesting condition is realised (cf. Ref. [2]). (b) Joint density of states (jDOS) of PdS_2 (red line) and PtS_2 (blue line). (c) Square modulus of the electric dipole matrix elements $|d_{cv\mathbf{k}}|^2$ for transitions $v \to c$, at point $\mathbf{k}$ in the irreducible Brillouin zone, contributing to the G (first column) and R (second column) excitons in PdS_2 (top row) and PtS_2 (bottom row).

References

[1] T. Sohier, M. Calandra, F. Mauri, *Phys. Rev. B* **96**, 075448 (2017).

[2] A. Carvalho, R. M. Ribeiro, A. H. Castro Neto, *Phys. Rev. B* **88**, 115205 (2013).
